# Noninflammatory obstructive appendicopathy: A rare presentation of abdominal pain

**DOI:** 10.1002/ccr3.7431

**Published:** 2023-05-26

**Authors:** Wissam Jamal Al Tamr, Sameh Ali, Kareem Omran

**Affiliations:** ^1^ NMC Royal Hospital Sharjah United Arab Emirates; ^2^ Department of Public Health and Primary Care University of Cambridge Cambridge UK

**Keywords:** appendicitis, emergency medicine, gastroenterology and hepatology, general surgery, pediatrics and adolescent medicine

## Abstract

Patients with appendicitis‐like abdominal pain but negative laboratory and radiological findings can have rare alternative aetiologies such as obstructive appendicopathy. A normal appendix may be seen in surgery, and removal with consent is curative.

## 1 INTRODUCTION

Acute abdominal pain is a common presenting symptom in emergency departments, accounting for a significant proportion of hospital admissions.^1^ The differential diagnosis of acute abdominal pain is broad and encompasses numerous gastrointestinal, genitourinary, and gynecological conditions.^2^ However, among the various causes of acute abdominal pain, appendicitis is one of the most frequent diagnoses, particularly in children and young adults.^3^, ^4^ Whilst Appendicitis is an inflammatory condition of the vermiform appendix, appendicopathy is a similar, but rare pathology of the vermiform appendix, mimicking the symptoms of appendicitis, but showing no histologic signs of inflammation.^5^

This case report describes the management of a case of obstructive appendicopathy due to follicular lymphoid hyperplasia with fecalith in a 12‐year‐old patient presenting to the hospital with an acute surgical abdomen and discusses the condition's relevance in pediatric surgery.

## 2 CASE HISTORY/EXAMINATION

A 12‐year‐old female presented to our institute with a history of intermittent, crampy abdominal pain for a duration greater than 10 days, which had significantly increased in severity over the previous week. She reported multiple episodes of non‐bilious vomiting the night before her presentation to our hospital but did not complain of urinary symptoms, constipation, or diarrhea over the duration of her symptoms. She denied experiencing fever, weight loss, or recent travel. She had presented to three different hospitals prior to this facility. Patient notes stated that ultrasonography, computed tomography (CT) scans, and routine bloods were performed. All suggested negative findings, and due to this appendicitis was ruled out, with the patient being discharged with analgesics.

During the physical examination at our clinic, the patient exhibited a stooped posture, abdominal kinking, and difficulty standing upright due to pain, which also affected her ability to walk. She had no fever, and her heart rate and blood pressure were within normal limits. The abdominal examination revealed significant tenderness in the right iliac fossa and a positive McBurney's sign, but there was no rebound tenderness or guarding. Rovsing's sign, psoas, and obturator signs were all negative. The remainder of her physical examination, including genitourinary and gynecological assessments, showed no abnormalities.

## 3 INVESTIGATIONS

Laboratory tests were performed and showed a normal blood profile, with a white blood cell count (WBC) of 6.9 × 10^9^/L, a normal C‐reactive protein (CRP) level (<4), and unremarkable liver and kidney function tests. A urinalysis was within normal limits, and a pregnancy test was negative.

Given the clinical suspicion of appendicitis, an abdominal ultrasound was requested. However, the examination did not reveal any abnormalities, and the appendix was not visualized. Due to the inconclusive ultrasound findings, a contrast‐enhanced CT scan of the abdomen was performed. The CT scan showed minimal fluid in the endometrial cavity, an appendix of average caliber with a diameter of 5.2 mm, and signs of mild hepatomegaly, without any radiological signs suggestive of appendicitis, such as appendiceal wall thickening, peri appendiceal fat stranding, or an appendicolith.

## 4 TREATMENT

**FIGURE 1:**
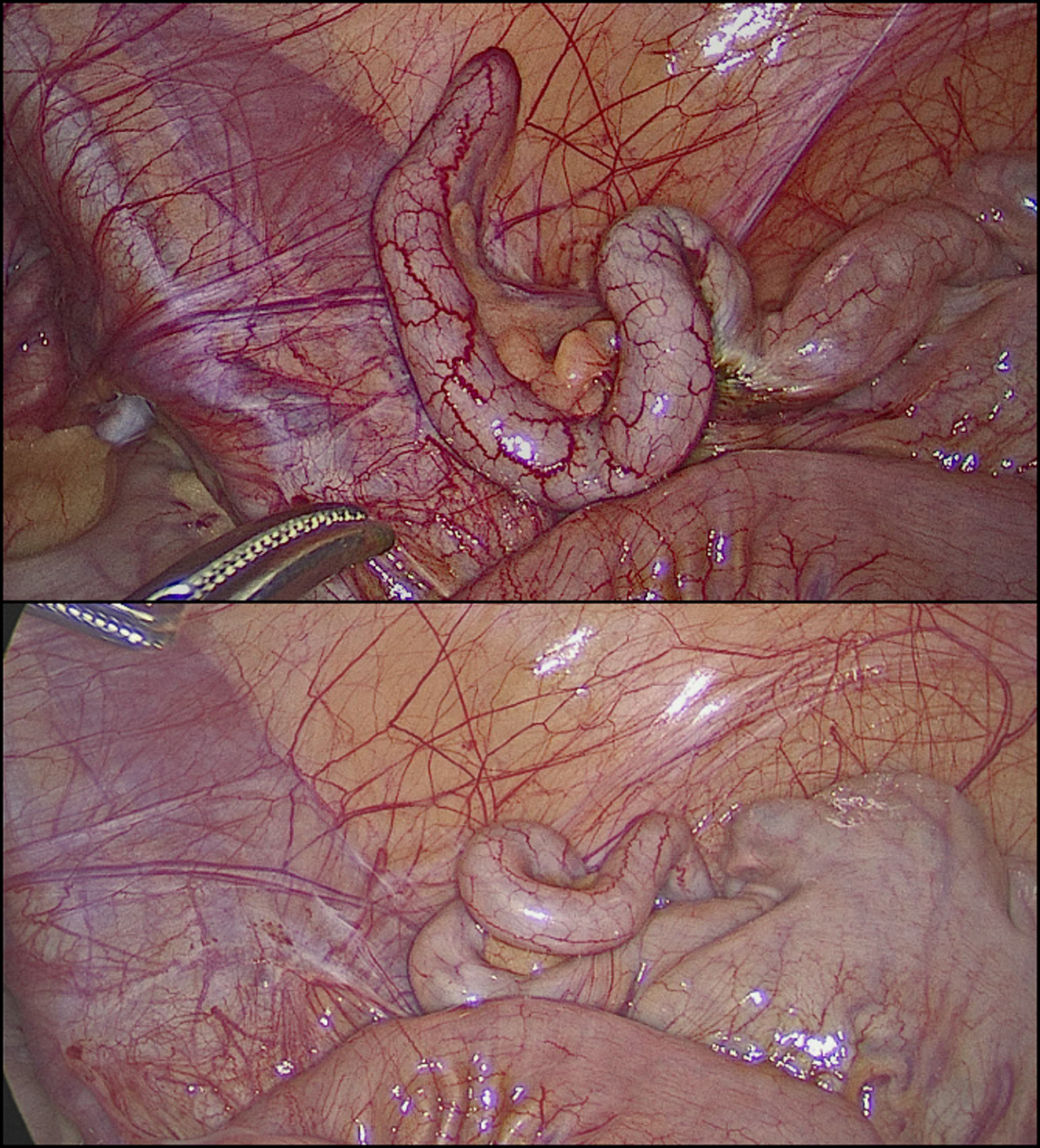
Macroscopically normal‐looking appendix discovered during diagnostic laparoscopy.

Considering the patient's persistent symptoms and the possibility of appendicitis, the decision was made to proceed with diagnostic laparoscopy – with the intention to remove the appendix if no other pathology was found. The patient's parents were informed about the diagnostic uncertainty and the potential for negative appendicitis and provided informed consent for the procedure. During the surgery, the appendix appeared grossly normal, without signs of inflammation, perforation, or abscess (Figure 1). Nevertheless, the appendix was removed to eliminate the possibility of underlying pathology, due to the very suggestive clinical examination findings, and sent to the laboratory for histopathological examination.

Following the surgery, the patient was meticulously monitored, and her pain was effectively managed using analgesics. Remarkably, within just a few hours post‐surgery, she exhibited a rapid and impressive recovery, as her pain subsided, and she regained the ability to walk with ease. By the second day after surgery, the patient's clinical condition was stable, she experienced no pain and had successfully resumed tolerating oral intake. Consequently, she was discharged from the hospital. Over the course of 4 months, the patient attended three post‐operative follow‐up visits, all of which confirmed a normal and complete recovery.

## 5 HISTOPATHOLOGY

**FIGURE 2:**
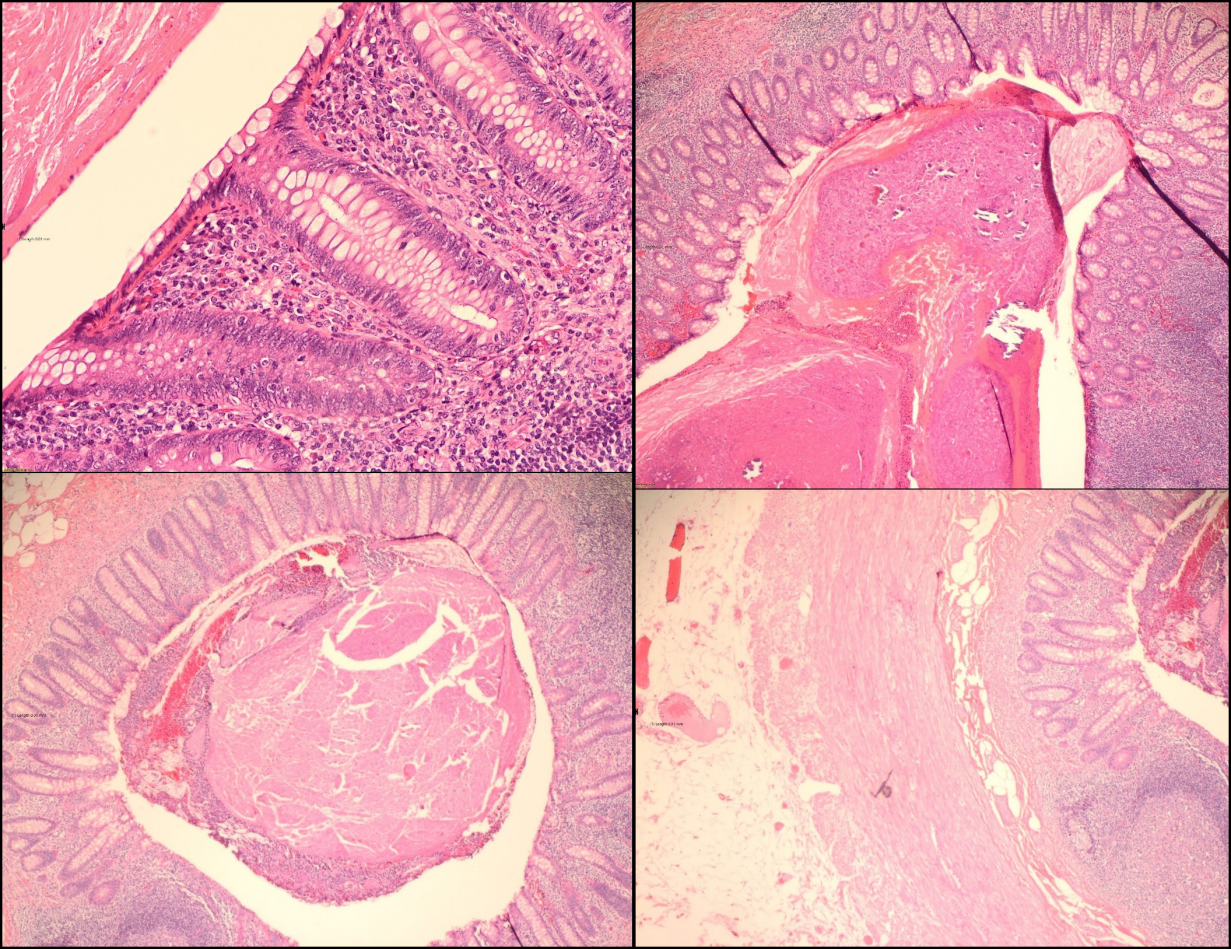
Histopathology slides revealing lymphoid hyperplasia of the mucosa and submucosa, with a slightly dilated appendiceal lumen filled with fecalith, but no evidence of an inflammatory reaction.

The appendix was taken to histopathology. Gross examination revealed an appendix measuring 7 × 7 × 0.5 cm with attached fat measuring 6 × 1 cm. Microscopy revealed a slightly dilated appendiceal lumen filled with fecalith with lymphoid follicular hyperplasia of the mucosa and submucosa. There was no evidence of acute inflammatory response. This pointed towards a diagnosis of follicular lymphoid hyperplasia with fecalith, consistent with obstructive appendicopathy (Figure 2).

## 6 DISCUSSION

Acute appendicitis ranks among the prevalent causes of acute abdominal pain in children and stands as the leading surgical cause of acute abdomen.^3^, ^4^ Inflammation serves as one of the hallmarks of this condition, leading to a rise in inflammatory markers, such as increased CRP levels and elevated WBCs.^6^ These are often accompanied by symptoms due to inflammation, such as Rovsing sign and rebound tenderness.^6^ In exceedingly rare cases, appendiceal pathology may present without any signs of inflammation, as illustrated by the index patient in this study. More frequently, such cases have been attributed to neurogenic appendicopathy—a histological diagnosis characterized by an increased number of neurofibers in the appendix and an absence of acute inflammation.^7^ Another, even rarer, etiology of appendicopathy is “obstructive appendicopathy,” which also lacks signs of inflammation but also has no neurological signs on histology.^5^ With no indications of scarring (which would suggest chronic inflammation) or acute inflammation, this condition is hypothesized to be a congenital anomaly.^5^

Non‐inflammatory aetiologies are less commonly encountered and can pose diagnostic challenges, especially when the clinical presentation is atypical or when laboratory and radiological findings are inconclusive. The importance lies in the high‐risk consequence of their misdiagnosis, as they may rupture and proceed to presume a clinical emergency. In this case, the patient visited three hospitals prior to admission to our facility, and patient notes indicated that appendicitis was ruled out on all three occasions due to the negative radiological and laboratory findings. The atypical presentation of acute abdominal pain without any laboratory or radiological markers was ultimately diagnosed as non‐inflammatory appendicopathy, likely due to fecalith obstruction secondary to follicular lymphoid hyperplasia.^8^ The missed diagnosis in this patient on multiple occasions underscores the importance of the knowledge and consideration of alternative causes of appendiceal pathology.

Accurate diagnosis of appendiceal pathology relies on a comprehensive assessment that integrates patient history, physical examination, laboratory tests, and imaging studies such as ultrasound and CT scans. Although ultrasound and CT scans are effective in diagnosing appendicitis, with reported accuracy rates of 71%–97% and 93%–98%, respectively,^9^ their results may not always be definitive.^10^ In some cases, such as this, even after performing diagnostic laparoscopy, the diagnosis may remain inconclusive due to the macroscopic appearance of an unaffected appendix, posing challenges in making surgical decisions regarding resection.^11^

Research indicates that although instances of acute right iliac fossa pain without accompanying biological signs of inflammation are uncommon in appendicitis, CT scans and ultrasonography are highly beneficial for accurate diagnosis in such cases.^12^ Furthermore, various studies have suggested that when WBCs and CRP values are within the normal range, the likelihood of acute appendicitis is low, and it is generally safe to discharge the patient.^13^, ^14^, ^15^ However, our index patient's case deviated from these expectations as the appendix was not visualized on ultrasound, and the CT scan revealed only mild hepatomegaly with the appendix appearing normal. Discharging the patient in this instance would have been dangerous, as studies have demonstrated that time is the primary driving factor contributing to disease progression and perforation in obstructive appendiceal pathology.^16^, ^17^, ^18^, ^19^ The lack of awareness and need for prompt management further underscores the importance of this case.

The management of cases like these can be particularly challenging, as even after diagnostic laparoscopy, the appendix may appear normal. The potential negative physical findings of the appendix during surgery emphasize the importance of ruling out other major causes of acute abdomen before diagnostic laparoscopy and the importance of awareness about such conditions. It is therefore crucial to discuss the high possibility of negative appendicitis with the guardians and acknowledge that the patient might still experience pain if the removal of the appendix does not address the root cause. Informed consent and adequate consultation with the patient and guardians are hence a cornerstone in the management of this condition. Nevertheless, when suspicion is high, the benefits of prompt surgical intervention outweigh the risks. Timely removal of the appendix can potentially avert life‐threatening complications associated with appendiceal obstruction, such as perforation, abscess formation, or peritonitis.^20^

## AUTHOR CONTRIBUTIONS

**Wissam Jamal Al Tamr:** Conceptualization; investigation; methodology; resources; supervision; writing – review and editing. **Sameh Ali:** Conceptualization; resources; supervision; writing – review and editing. **Kareem Omran:** Conceptualization; data curation; investigation; methodology; writing – original draft; writing – review and editing.

## FUNDING INFORMATION

None.

## CONFLICT OF INTEREST STATEMENT

The authors declare no conflicts of interest.

## CONSENT

Written informed consent was obtained from the patient to publish this report in accordance with the journal's patient consent policy.

## Data Availability

Data sharing is not applicable to this article as no new data were created or analyzed in this study.

## References

[1] Dadeh AA . Factors associated with unfavorable outcomes in patients with acute abdominal pain visiting the emergency department. BMC Emerg Med. 2022;22(1):195. 36474160 10.1186/s12873-022-00761-y PMC9727909

[2] Mehta H . Abdominal pain. In: David, S. (Ed.), Clinical Pathways in Emergency Medicine. Springer; 2016. doi:10.1007/978-81-322-2710-6_26

[3] Ross A , LeLeiko NS . Acute abdominal pain. Pediatr Rev. 2010;31(4):135‐144. 20360407 10.1542/pir.31-4-135

[4] Carty H . Paediatric emergencies: non‐traumatic abdominal emergencies. Eur Radiol. 2002;12:2835‐2848. 12439562 10.1007/s00330-002-1499-7

[5] Antonakopoulos GN , Edwards C , Panayiotides JG . Obstructive appendicopathy: a disease or a congenital entity? J Clin Pathol. 2008;61(7):874‐875. 18587021 10.1136/jcp.2007.054213

[6] Jones MW , Lopez RA , Deppen JG . Appendicitis. StatPearls [Internet]. StatPearls Publishing; 2021. https://www.ncbi.nlm.nih.gov/books/NBK493193/

[7] Partecke LI , Thiele A , Schmidt‐Wankel F , et al. Appendicopathy—a clinical and diagnostic dilemma. Int J Colorectal Dis. 2013;28(8):1081‐1089. 23516073 10.1007/s00384-013-1677-x

[8] Daldrup‐Link HE . Lymphoid follicular hyperplasia. In: Newman B , Daldrup‐Link HE , eds. Pearls and pitfalls in pediatric imaging: variants and other difficult diagnoses. Cambridge University Press; 2014:215‐217.

[9] Old JL , Dusing RW , Yap W , Dirks J . Imaging for suspected appendicitis. Am Fam Physician. 2005;71(1):71‐78. 15663029

[10] Crocker C , Akl M , Abdolell M , Kamali M , Costa AF . Ultrasound and CT in the diagnosis of appendicitis: accuracy with consideration of indeterminate examinations according to STARD guidelines. AJR Am J Roentgenol. 2020;215(3):639‐644. 32406773 10.2214/AJR.19.22370

[11] Partecke LI , von Bernstorff W , Karrasch A , et al. Unexpected findings on laparoscopy for suspected acute appendicitis: a pro for laparoscopic appendectomy as the standard procedure for acute appendicitis. Langenbecks Arch Surg. 2010;395:1069‐1076. 19924435 10.1007/s00423-009-0567-8

[12] Monneuse O , Abdalla S , Pilleul F , et al. Pain as the only consistent sign of acute appendicitis: lack of inflammatory signs does not exclude the diagnosis. World J Surg. 2010;34(2):210‐215. 20041246 10.1007/s00268-009-0349-z

[13] Sengupta A , Bax G , Paterson‐Brown S . White cell count and C‐reactive protein measurement in patients with possible appendicitis. Ann R Coll Surg Engl. 2009;91(2):113‐115. 19102827 10.1308/003588409X359330 PMC2749345

[14] Arfa N , Gharbi L , Marsaoui L , et al. Value of admission for observation in the management of acute abdominal right iliac fossa pain. Prospective study of 205 cases. Press Med. 2006;35(3 Pt 1):393‐398. 10.1016/s0755-4982(06)74602-4 16550128

[15] Yang HR , Wang YC , Chung PK , Chen WK , Jeng LB , Chen RJ . Laboratory tests in patients with acute appendicitis. ANZ J Surg. 2006;76(1–2):71‐74. 16483301 10.1111/j.1445-2197.2006.03645.x

[16] Drake FT , Mottey NE , Farrokhi ET , et al. Time to appendectomy and risk of perforation in acute appendicitis. JAMA Surg. 2014;149(8):837‐844. 24990687 10.1001/jamasurg.2014.77 PMC4160117

[17] Papandria D , Goldstein SD , Rhee D , et al. Risk of perforation increases with delay in recognition and surgery for acute appendicitis. J Surg Res. 2013;184(2):723‐739. 23290595 10.1016/j.jss.2012.12.008 PMC4398569

[18] Busch M , Gutzwiller FS , Aellig S , Kuettel R , Metzger U , Zingg U . In‐hospital delay increases the risk of perforation in adults with appendicitis. World J Surg. 2011;35:1626‐1633. 21562871 10.1007/s00268-011-1101-z

[19] Kearney D , Cahill R , O'brien E , Kirwan W , Redmond H . Influence of delays on perforation risk in adults with acute appendicitis. Dis Colon Rectum. 2008;51:1823‐1827. 18584252 10.1007/s10350-008-9373-6

[20] Makama JG , Kache SA , Ajah LJ , Ameh EA . Intestinal obstruction caused by appendicitis: a systematic review. J West Afr Coll Surg. 2017;7(3):94‐115. 30525005 PMC6237405

